# Supplementary material for: Not so sluggish: the success of the Felimare picta complex (Gastropoda, Nudibranchia) crossing Atlantic biogeographic barriers
Source: PeerJ. 2016 Jan 19;4:e1561. doi: 10.7717/peerj.1561 (PMC4730986; doi:10.7717/peerj.1561)
Supplement: Supplemental Information 1 — Table S1–Distribution areas of the six subspecies described for Felimare (Hypselodoris) picta. [file peerj-04-1561-s001.docx]

| **Subspecies of *Felimare picta*** | **Distribution area** | **References** |
| --- | --- | --- |
| *Felimare picta picta* (Schultz, 1836) | Western Atlantic to the Mediterranean, south to Cape Verde Islands | Ortea, Valdés & García-Gómez (1996); Gosliner & Johnson (1999) |
| *Felimare picta webbi* (d'Orbigny, 1839) | West Atlantic from Florida to Brazil and East Atlantic from south of Spain to the Canary Islands | Ortea, Valdés & García-Gómez (1996) |
| *Felimare picta tema* Edmunds, 1981 | West Africa (Ghana) | Edmunds (1981); Ortea, Valdés & García-Gómez (1996) |
| *Felimare picta verdensis* (Ortea, Valdés & Garcia-Gomez, 1996) | Archipelagos of Cape Verde and S. Tomé and also south Angola | Ortea, Valdés & García-Gómez (1996) |
| *Felimare picta azorica* (Ortea, Valdés & Garcia-Gomez, 1996) | Archipelago of the Azores | Ortea, Valdés & García-Gómez (1996) |
| *Felimare picta lajensis* Troncoso, Garcia & Urgorri, 1998 | Southwest Atlantic (south and southeastern of Brazil) | Domínguez, García & Troncoso (2006); Dacosta, Padula & Schrödl (2010) |
