# Supplementary material for: Not so sluggish: the success of the Felimare picta complex (Gastropoda, Nudibranchia) crossing Atlantic biogeographic barriers
Source: PeerJ. 2016 Jan 19;4:e1561. doi: 10.7717/peerj.1561 (PMC4730986; doi:10.7717/peerj.1561)
Supplement: Supplemental Information 2 — Table S2–List of material included in this study, sampling localities and Genbank accession numbers. [file peerj-04-1561-s002.docx]

| **Species** | **Sampling site** |  | **Genbank accession numbers** | |
| --- | --- | --- | --- | --- |
|  |  | **Coordinates** | **16S** | **COI** |
| *Felimare picta picta* | Italy | 40°49’59’’N/014°15’10’’E | KT820489  KT820490 | KT833228  KT833229 |
|  |  |  |  | KT833230 |
|  | S Spain | 36°10’52’’N/006°2’10’’W | KT820491  KT820492 | KT833231  KT833232 |
|  |  | 36°0’35’’N/005°36’04’’W | KT820493 | KT833233 |
|  | SW Mediterranean | 35°10’47’’N/002°25’46’’W | KT820494 | KT833234  KT833235 |
|  | Portugal | 36°31’01’’N/011°34’01’’W | KT820495 KT820496  KT820497 | KT833236  KT833237 |
|  | Madeira | 30°08’26’’N/015°52’10’’W | KT820498 | KT833238 |
|  | Canary Islands | 28°3’39’’N/016°30’54’’W | KT820499  KT820500 | KT833239  KT833240 |
|  | Mexico | 20°38’39’’N/090°28’24’’W | KT820501 KT820502 | KT833241  KT833242  KT833243 |
| *Felimare picta azorica* | Azores | 37°42’50’’N/025°26’04’’W | KT820503 KT820504  KT820505 KT820506 | KT833244  KT833245  KT833246  KT833247 |
|  |  | 39°31’40’’N/031°12’24’’W | KT820507 | KT833248 |
|  |  | 38°31’27’’N/028°37’20’’W | KT820508 |  |
|  |  | 38°39’16’’N/027°13’49’’W | KT820509 KT820510 | KT833249  KT833250 |
| *Felimare picta verdensis* | São Tomé | 0°18’11’’N/006°45’22’’E | KT820511  KT820512 KT820513 | KT833251  KT833252 |
|  | Cape Verde | 16°52’53’’N/024°59’58’’W | KT820515 | KT833254 |
| *Felimare picta tema* | Senegal | 14°40’07’’N/017°23’53’’W | KT820514 | KT833253 |
| *Felimare zebra* | Bermuda | 32°16’36’’N/064°49’21’’W | KT820516 | KT833255 |
| *Felimare lajensis* | Brazil | 22°53’26’’S/042°0’24’’W | KT820517  KT820518 | KT833256 |
| *Felimare acriba* | Mexico | 21°21’55’’N/089°9’24’’W | KT820519 |  |
| *Felimare bayeri* | Mexico | 18°36’07’’N/092°42’23’’W | KT820520 | KT833257 |
| *Felimare ruthae* | Mexico | 21°21’55’’N/089°9’24’’W | KT820521 | EU982747.1 |
| *Felimare kempfi* | Mexico | 21°21’55’’N/089°9’24’’W | KT820522 | EF535121.1 |
| *Felimare cantabrica* | NW Spain | 43°28’05’’N/008°16’36’’W | KT820523  KT820524 | KT833258 |
|  | NW Portugal | 40°38’25’’N/008°45’16’’W | KT820525 KT820526 |  |
| *Felimare villafranca* | NW Spain | 43°28’05’’N/008°16’36’’W | KT820527  KT820528 KT820529 | KT833260  KT833261  KT833262 |
| *Felimare fontandraui* | W Portugal | 38°25’57’’N/009°7’06’’W | KT820530 | KT833263 |
| *Felimare midatlantica* | Azores | 37°42’50’’N/025°26’04’’W | KT820531 KT820532 | KT833259 |
| *Felimare pinna* | Cape Verde | 16°52’53’’N/024°59’58’’W | KT820533 |  |
| *Felimare bilineata* | W Africa |  | EF534052.2 | EF535125.1 |
| *Felimare californiensis* | E Pacific |  | EU982796.1 | EU982744.1 |
| *Felimare orsinii* | W Mediterranean |  | AJ225189.1 | AJ223265.1 |
| *Hypselodoris infucata* | Indo W Pacific |  | FJ917426.1 | JQ727891.1 |
| *Hypselodoris obscura* | SE Australia |  | EU982797.1 | EF535130.1 |
| *Hypselodoris paulinae* | Hawaii |  | EU982798.1 | EU982746.1 |
| *Hypselodoris* *bullocki* | Indo W Pacific |  | EU982795.1 | EU982743.1 |
| *Hypselodoris bennetti* | SE Australia |  | EF534059.2 | EF535131.1 |
| *Chromodoris krohni* | Azores | 37°42’50’’N/025°26’04’’W | KT820535 | KT833264 |
|  | S Portugal | 36°59’48’’N/008°56’38’’W | KT820534 | KT833265 |
| *Doriopsilla pelseneeri* | W Portugal | 39°24’40’’N/009°30’38’’W | KT820536 | KT833266  KT833267 |
