# Supplementary material for: Not so sluggish: the success of the Felimare picta complex (Gastropoda, Nudibranchia) crossing Atlantic biogeographic barriers
Source: PeerJ. 2016 Jan 19;4:e1561. doi: 10.7717/peerj.1561 (PMC4730986; doi:10.7717/peerj.1561)
Supplement: Supplemental Information 3 — Diagnostic nucleotide characteres for the 16S and COI DNA fragments listed for F. picta (including former F. picta picta, F picta webbi and F. picta azorica), F. lajensis, F. zebra and F. tema. [file peerj-04-1561-s003.docx]

**16S** 20 23 77 106 116 124 128 149 168 169 213 221 232 258 261 263 268 269 270 288 307

*F. picta* (22) TTATACCT..A..T..T...C...G...T...A...T...A...G...G...A...A...Y...T...T...G...G...A...C...Y

*F. lajensis* (2) TTATACCT..G..C..T...T...A...T...G...C...A...G...A...A...G...A...A...T...A...G...C...C...T

*F. zebra* (1) TTATACCT..G..T..C...T...A...T...A...T...A...G...A...G...G...A...A...T...A...G...C...C...T

*F. tema* (5) TTATACCT..A..T..T...T...A...C...A...T...G...A...A...A...A...-...G...C...A...A...C...T...G

**COI 03 09 24 27 30 54 63 69 70 87 93 123 129 132 144 159 171 180 183 198 201 213 216 225 231**

*F. picta* (23) TGA..T..Y..A..T..G..A..C..T..T..C...A...T...C...T...A...A...T...T...G...C...G...T...C...A

*F. lajensis* (1) TGG..T..T..G..A..A..G..C..C..T..T...A...C...C...T...G...A...C...C...G...T...G...T...C...A

*F. zebra* (1) TGA..T..T..A..A..A..G..T..C..C..T...A...C...C...T...A...G...C...C...G...T...G...T...T...C

*F. tema* (4) TGG..C..A..G..T..A..A..T..T..T..T...G...T...T...C...A...A...T...T...A...C...A...A...T...A

**234 237 258 261 273 288 291 294 297 312 315 318 327 339 360 361 372 378 393 400 417 427 442 463 472 476**

...T...G...C...G...C...C...T...G...A...G...T...T...C...T...T...C...T...T...T...C...-...T...A...G...T...C

...T...G...T...A...T...T...C...G...A...A...C...C...T...C...T...T...T...C...T...T...-...C...A...G...T...T

...T...G...T...A...T...T...A...G...A...G...T...T...T...C...T...C...C...C...T...T...A...C...A...G...T...T

...C...A...T...A...T...T...T...T...G...G...T...C...T...T...C...T...T...T...C...T...-...T...G...A...C...T

**481 484 489 493 505 510 520 535 540 544**

...C...A...G...A...C...C...A...C...T...-

...T...A...A...A...T...T...A...T...T...-

...T...A...A...A...T...T...A...T...T...T

...T...T...G...G...T...T...G...T...C...-
