# Supplementary figures and images for: Not so sluggish: the success of the Felimare picta complex (Gastropoda, Nudibranchia) crossing Atlantic biogeographic barriers
Source: PeerJ. 2016 Jan 19;4:e1561. doi: 10.7717/peerj.1561 (PMC4730986; doi:10.7717/peerj.1561)

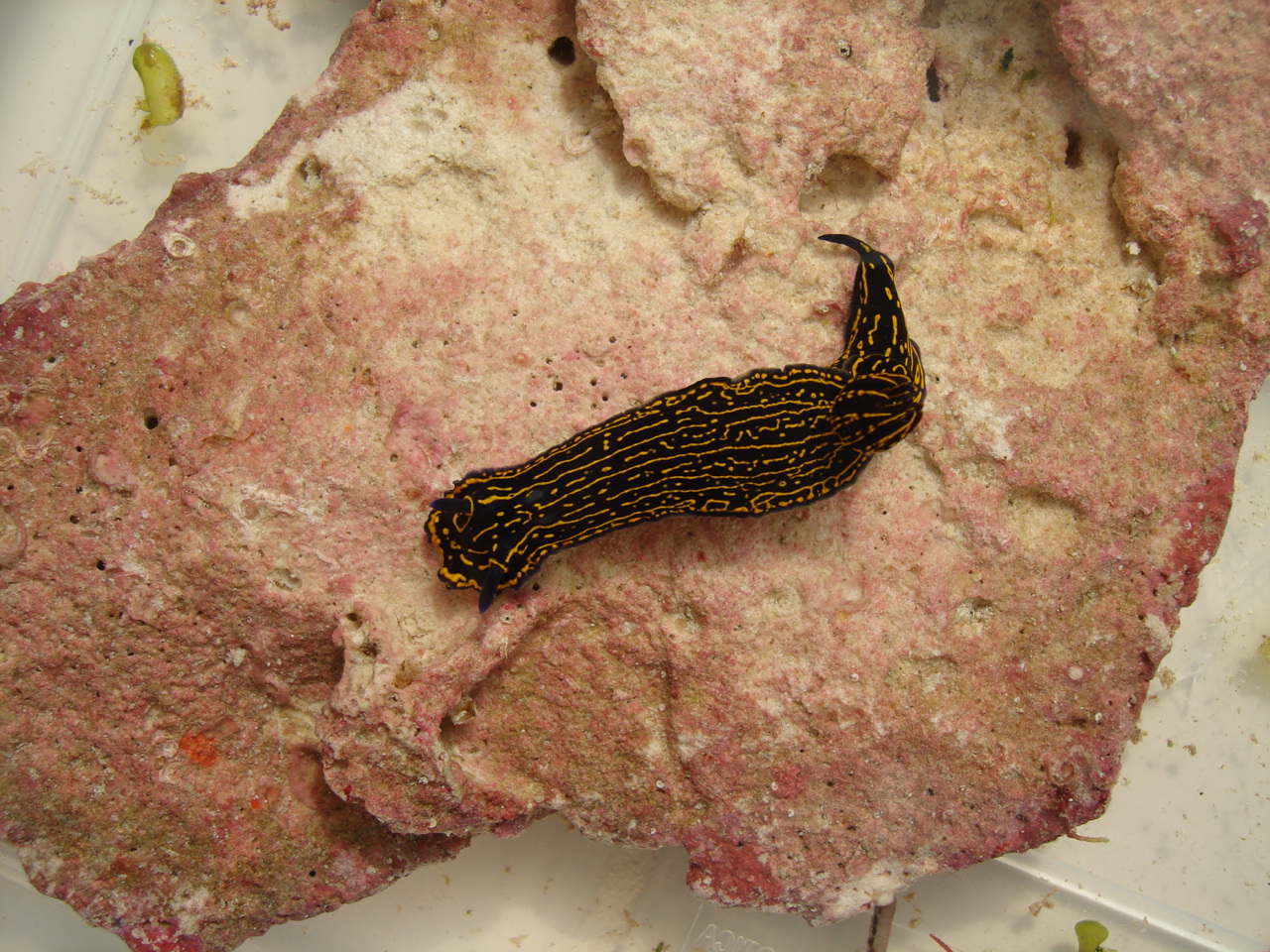

Supplement: Supplemental Information 4 — Felimare picta (Mexico). Credits to G. Calado. [file peerj-04-1561-s004.jpg]

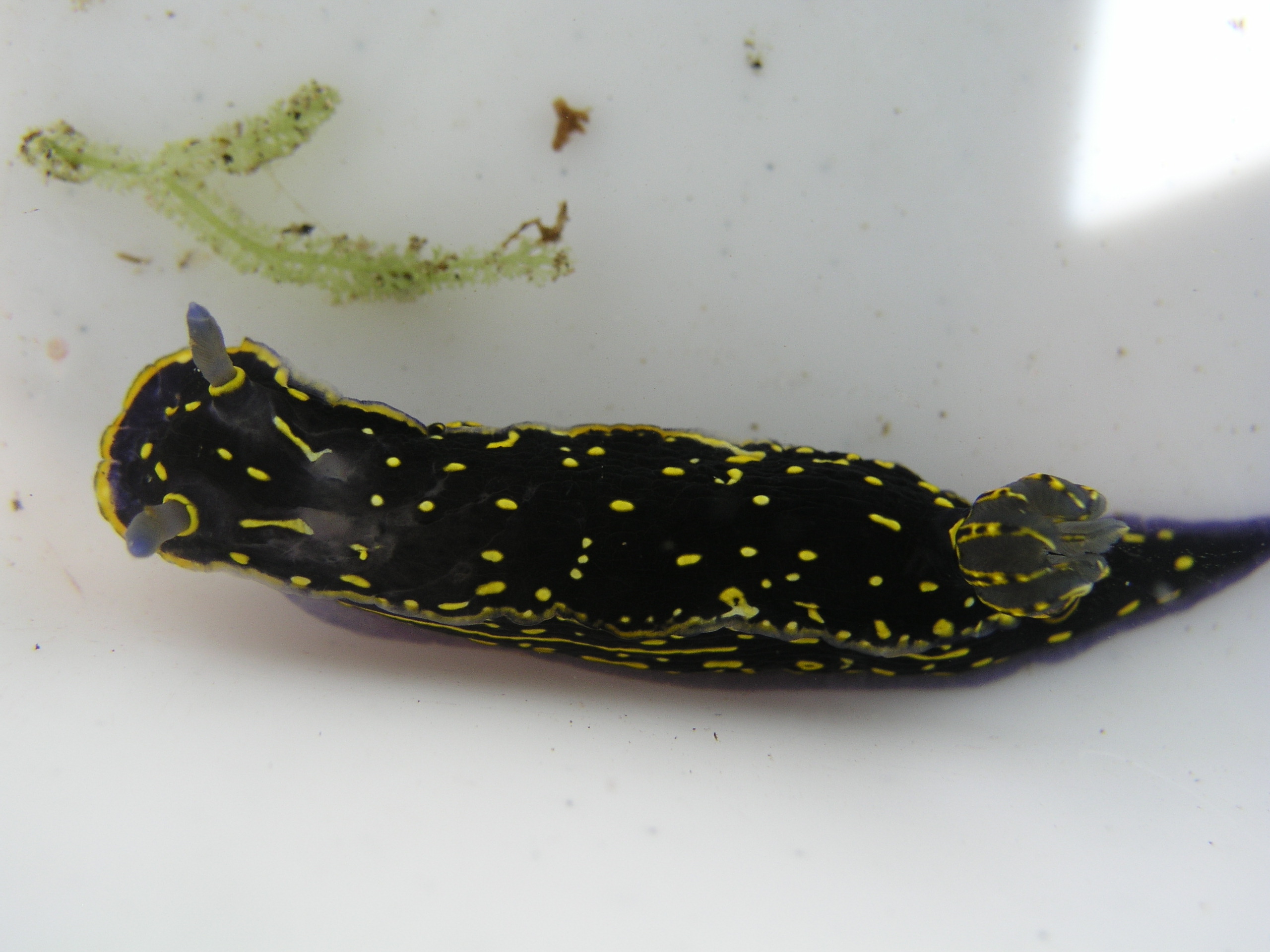

Supplement: Supplemental Information 5 — Felimare picta (Archipelago of the Azores). Credits to G. Calado. [file peerj-04-1561-s005.jpg]

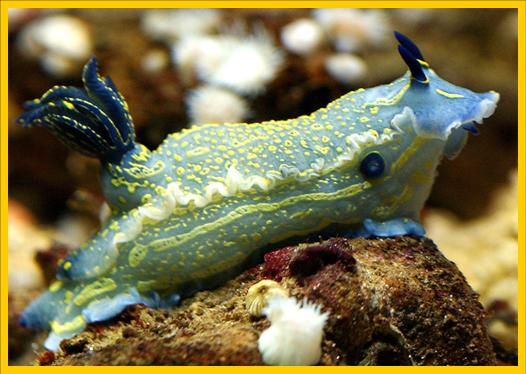

Supplement: Supplemental Information 6 — Felimare picta (South Portugal). Credits to R. Coelho/IPM. [file peerj-04-1561-s006.jpg]

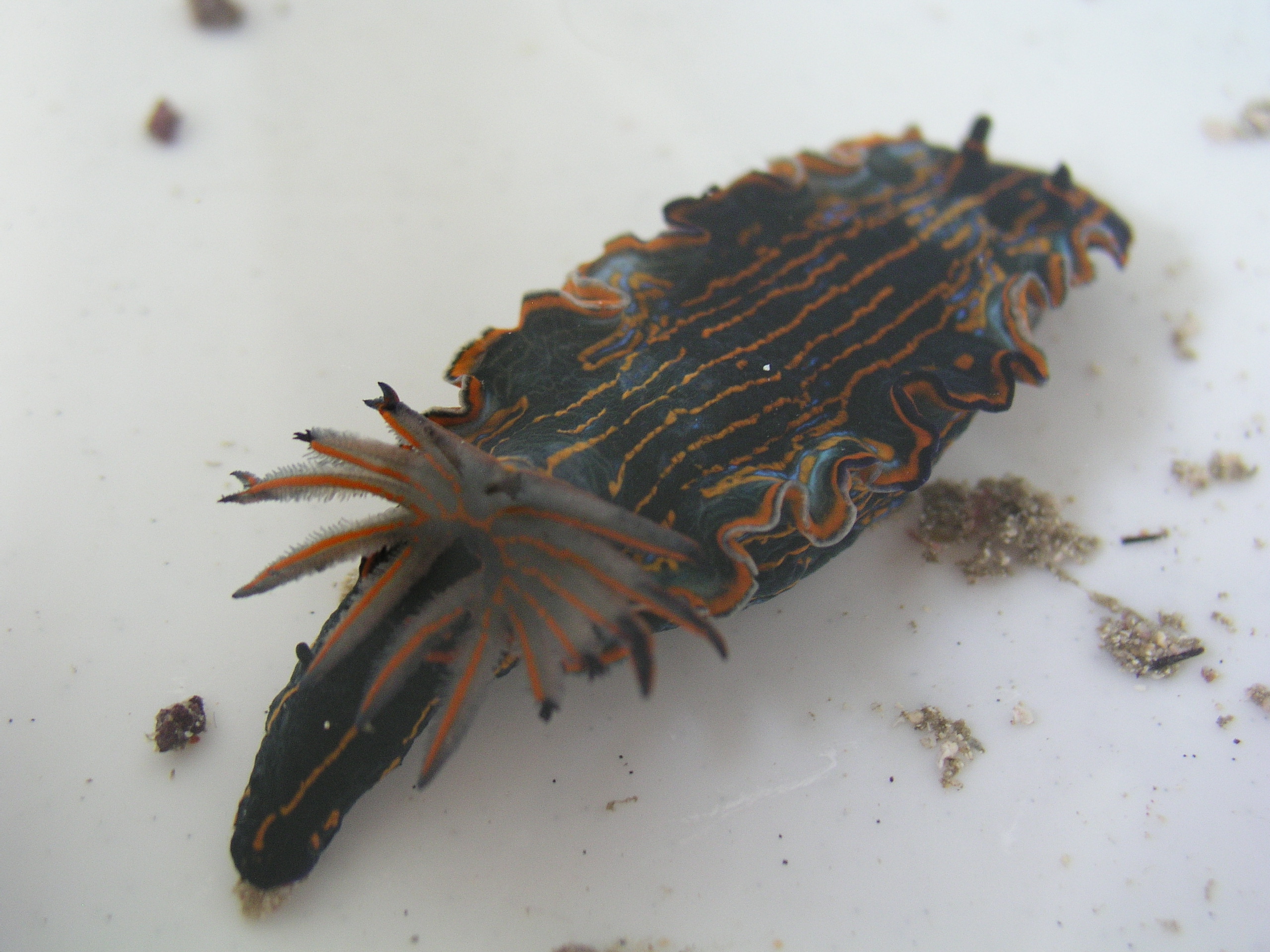

Supplement: Supplemental Information 7 — Felimare tema (S. Tome and Princepe). Credits to G. Calado. [file peerj-04-1561-s007.jpg]

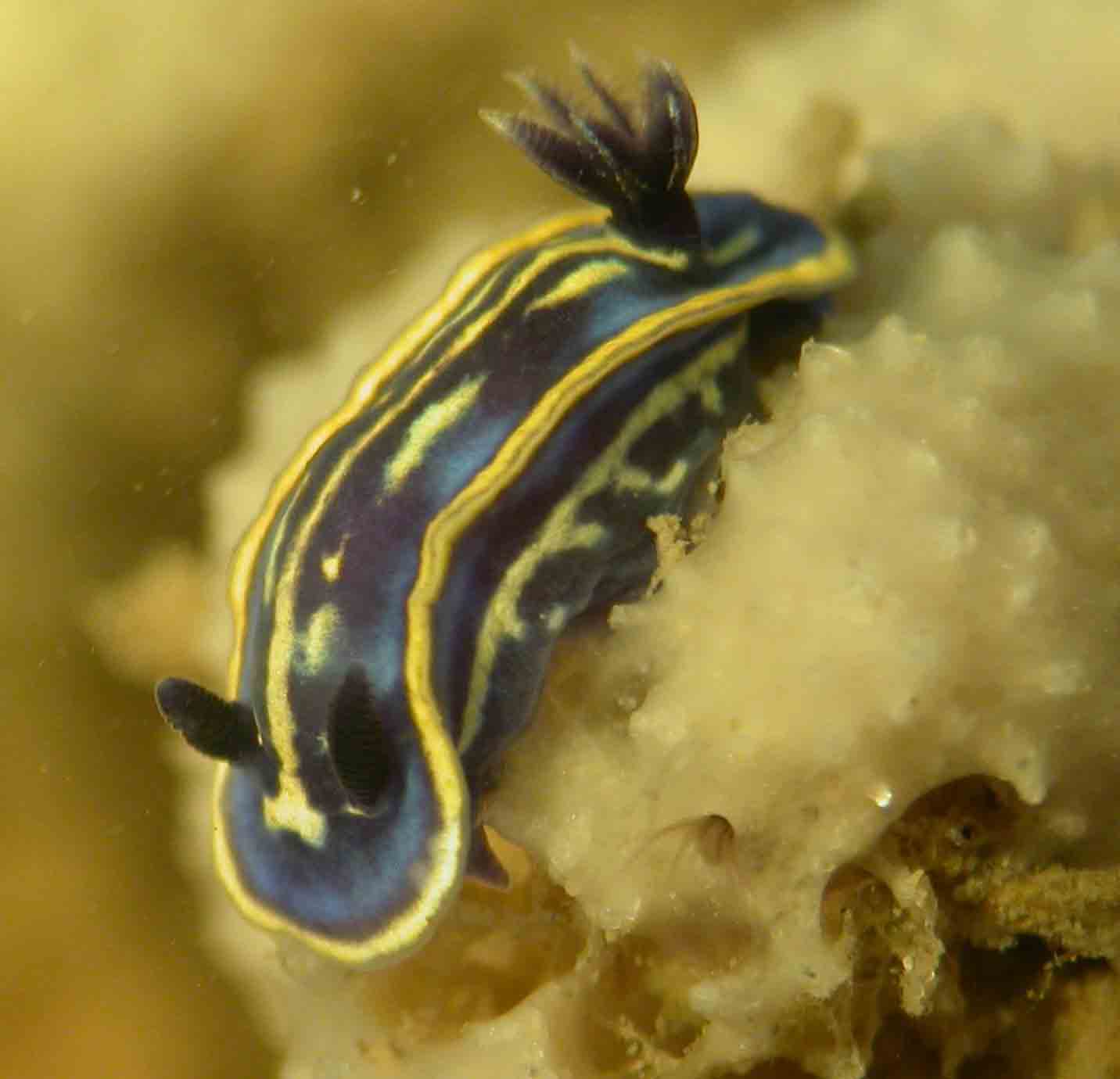

Supplement: Supplemental Information 8 — Felimare cantabrica (South Portugal). Credits to R. Coelho/IPM. [file peerj-04-1561-s008.jpg]

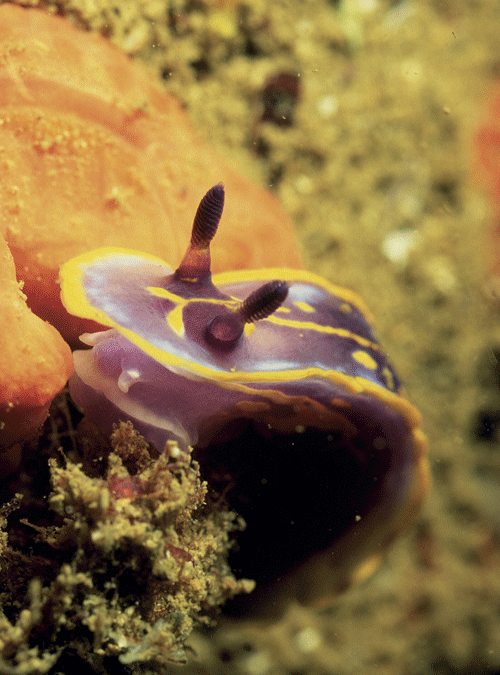

Supplement: Supplemental Information 9 — Felimare fontandraui (West Portugal). Credits to G. Calado. [file peerj-04-1561-s009.gif]

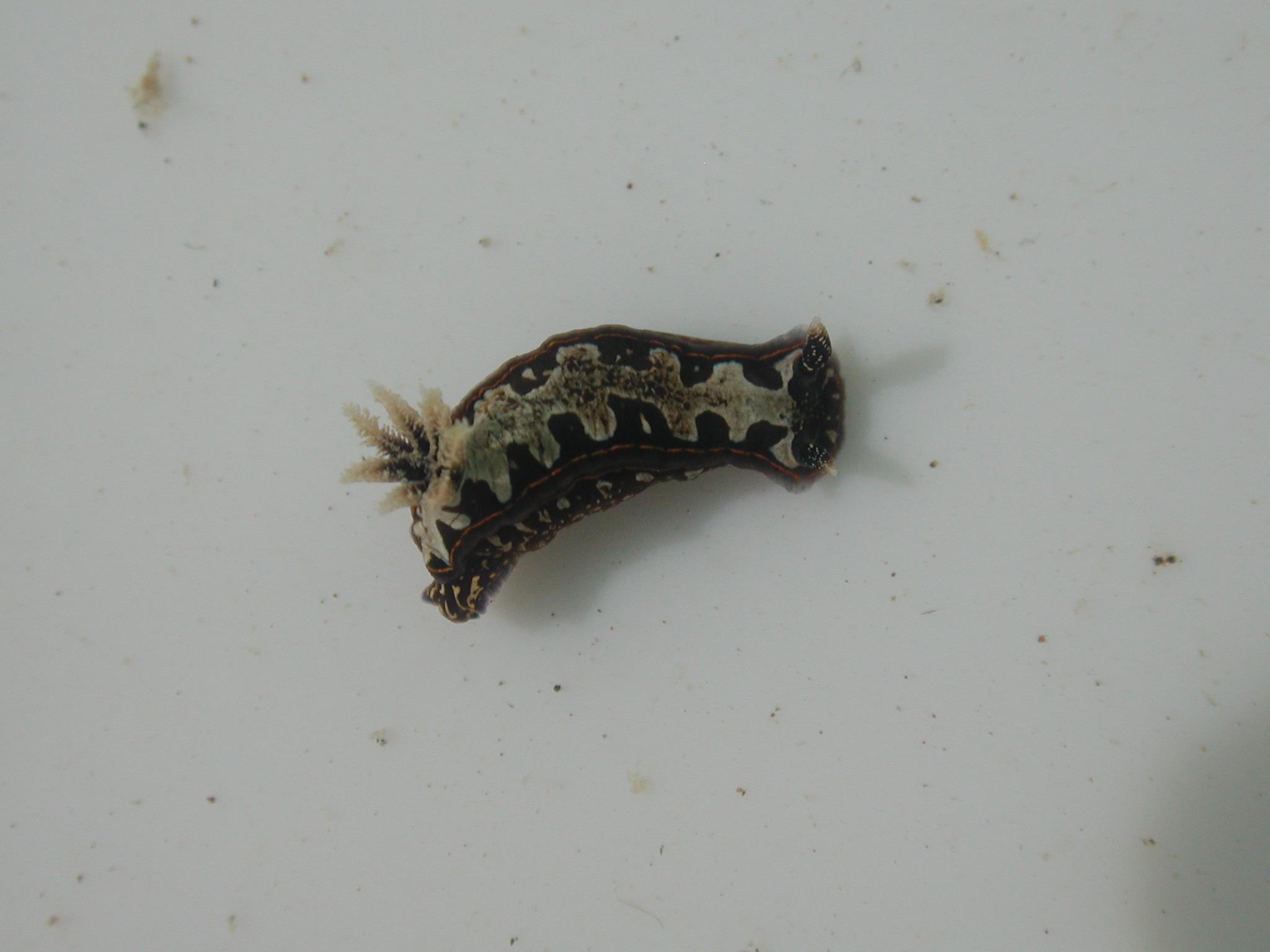

Supplement: Supplemental Information 10 — Felimare pinna (Cape Verde). Credits to G. Calado. [file peerj-04-1561-s010.jpg]

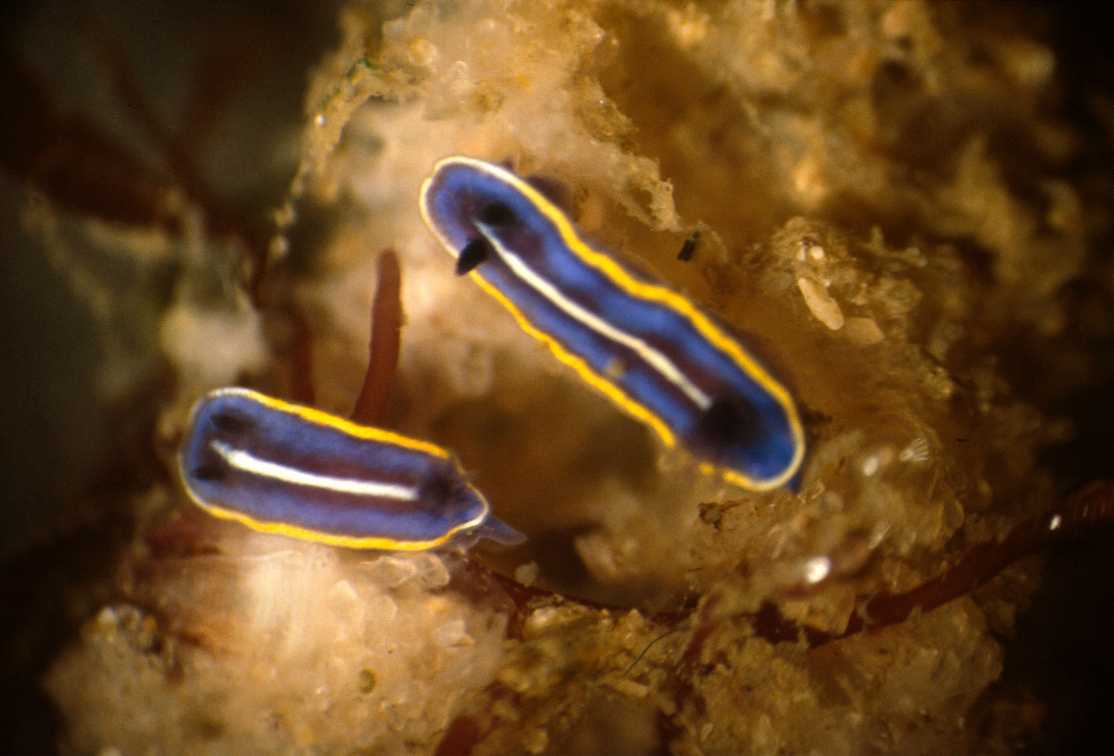

Supplement: Supplemental Information 11 — Felimare midatlantica (South Portugal). Credits to R. Coelho/IPM. [file peerj-04-1561-s011.jpg]

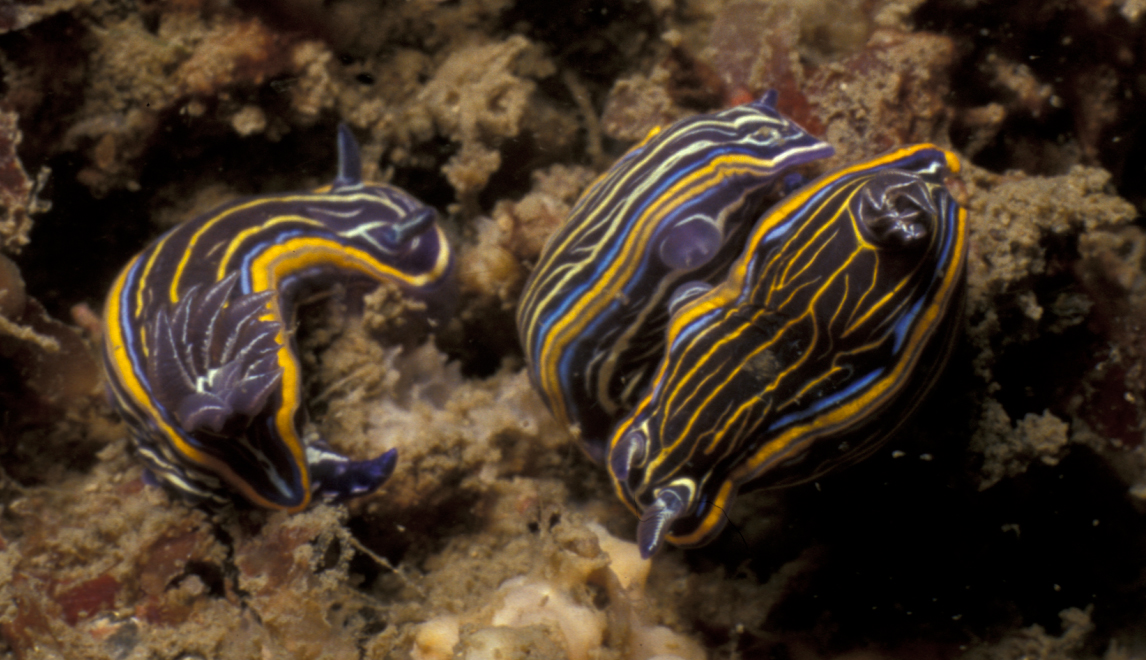

Supplement: Supplemental Information 12 — Felimare villafranca (South Portugal). Credits to R. Coelho/IPM. [file peerj-04-1561-s012.jpg]

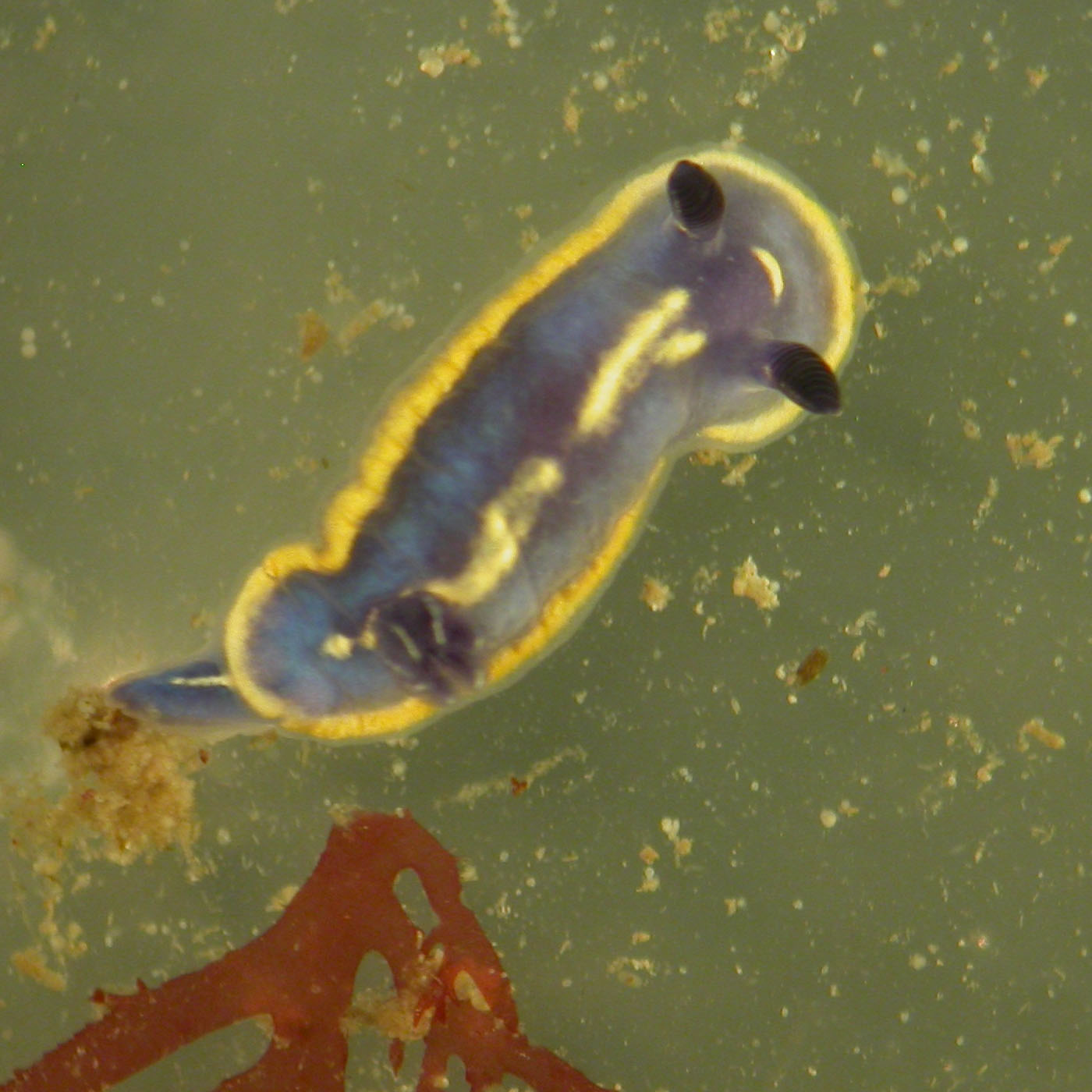

Supplement: Supplemental Information 13 — Felimare bilineata (South Portugal). Credits to R. Coelho/IPM. [file peerj-04-1561-s013.jpg]
